# Serum biomarkers to predict hemorrhagic transformation and ischemic stroke outcomes in a prospective cohort study

**DOI:** 10.25122/jml-2023-0148

**Published:** 2023-06

**Authors:** Elena Costru-Tasnic, Mihail Gavriliuc, Elena Manole

**Affiliations:** 1 Neurology Department no. 1, Nicolae Testemitanu State University of Medicine and Pharmacy, Chisinau, Republic of Moldova; 2 Diomid Gherman Institute of Neurology and Neurosurgery, Chisinau, Republic of Moldova

**Keywords:** biomarkers, hemorrhagic transformation, ischemic stroke prognosis, MMP-2, MMP-9, INR, AF: atrial fibrillation, CT: computed tomography, CRP: C-reactive protein, HT: hemorrhagic transformation, HDL: high-density lipoprotein, INR: International Normalised Ration, IS: ischemic stroke, LDL: low-density lipoprotein, MRI: magnetic resonance imaging, MMPs: matrix metalloproteinases, mRS: modified Rankin scale, NIHSS: National Institutes of Health Stroke Scale, NLR: neutrophils to lymphocytes ratio, TJPs: tight junction proteins

## Abstract

Ischemic stroke (IS) remains one of the most frequent causes of death and disability worldwide. Identifying possible prognosis factors for IS outcomes, including hemorrhagic transformation (HT), could improve patients' recovery. This study aimed to investigate the potential prognosis role of non-specific laboratory data at admission and baseline MMP-2 and MMP-9 serum levels in predicting HT risk, discharge, and 3-month follow-up status of IS patients. Data from 150 successive acute cerebral infarction patients were analyzed in a prospective cohort study. The active group included patients who developed HT during hospitalization (55 persons). There were no significant differences in age, gender distribution, time to admission, or time to blood sample collection for MMPs measurement between patients in the active and control groups. IS patients from the active group had a significantly higher rate of AF (atrial fibrillation) in the past (p=0.003), while differences in other factors such as diabetes, hypertension, myocardial infarction, previous stroke, obesity, smoking, and alcohol were not significant. Admission NIHSS score and mRS (modified Rankin Scale) values (at discharge and 90 days) were significantly worse in the active group (p<0.001). Among the analyzed admission laboratory factors (glycemia, lipid profile, coagulation panel, inflammatory reaction parameters, MMP-2, MMP-9), INR presented an inverse correlation, with lower values in the HT cohort (univariate analysis – p=0.01, OR=0.11; multivariate analysis - p=0.03, OR=0.09). Further research on larger cohorts is warranted to determine the specific laboratory biomarkers for predicting hemorrhagic transformation and ischemic stroke outcomes.

## INTRODUCTION

Ischemic stroke remains one of the most frequent causes of death and disability worldwide [1], placing a significant burden on patients and their families, and society as a whole. The actuality of stroke research is driven by the alarming trend of increasing incidence and prevalence rates [2] in the context of heterogeneity of risk factors and multiple stroke mimics [3], but also of limited time window available to save the affected brain parenchyma affected by the reduced blood supply [4]. Moreover, the paucity of time and treatment options for cerebral vessel recanalization faces numerous contraindications and complications, with hemorrhagic transformation (HT) being one the most feared side effect of both chemical and mechanical thrombolysis [5]. HT incidence is highly variable, depending on the type of study conducted. Radiologic and morphologic research studies have reported HT incidences as high as 70%, whereas clinical studies have reported lower rates, around 27%. However, the percentage of symptomatic cases remains relatively constant at around 5-6% [6–8]. An individualized and personalized approach to identifying possible prognosis factors for poor outcomes in ischemic stroke patients could minimize the risk for HT. Therefore, it is particularly important to find hallmarks or signatures of normal and pathological processes which could improve the management of the disease.

The signatures of normal or pathological states are called biomarkers, originally defined as specific characteristics or measurements that indicate normal biological processes, pathogenic processes, or responses to various exposures or interventions [9]. Biomarkers could be used both as diagnosis, but also prognosis tools. Myocardial infarction is a well-known example where increased troponin levels are used for diagnosis, and the dynamics of serum troponins reflect disease progression and response to treatment. In contrast, although ischemic stroke involves restricting blood supply to the brain, no specific biomarkers comparable to troponins have been identified to date for the diagnosis and prognosis of ischemic stroke patients. This can be attributed to the complex and diverse underlying pathological mechanisms contributing to ischemic stroke. Numerous laboratory parameters have been analyzed in both fundamental and clinical studies, including admission glycemia [10], serum lipids profile [11], as well as markers of inflammatory response such as C-reactive protein (CRP), leukocyte count, and neutrophil-to-lymphocyte ratio [12]. Additionally, specific proteins associated with neuronal injury (e.g., neuron-specific enolase, S100B) and blood-brain barrier disruption (e.g., tight junction proteins, matrix metalloproteinases, cellular fibronectin) have been investigated, with variable sensibility and specificity, indicating the necessity of further research in the field [13–16].

Among specific proteins, matrix metalloproteinases (MMPs) showed promising results in fundamental research with successful translation in clinical trials. Elevated levels of MMP-9 at admission have been strongly associated with stroke outcomes, including the prognosis for hemorrhagic transformation [17,18], and MMP-2 presents a particular association with cardioembolic stroke and its outcome [19].

The aim of our research was to investigate the potential prognostic role of non-specific laboratory parameters at admission, as well as baseline serum levels of MMP-2 and MMP-9 in predicting the risk of hemorrhagic transformation, discharge outcomes, and the 3-month follow-up status of patients with ischemic stroke.

## MATERIAL AND METHODS

We conducted a prospective cohort study to compare patients with acute ischemic stroke who experienced subsequent hemorrhagic transformation during hospitalization (active group) with those who did not (control group). The secondary outcome was to compare the mortality rate and functional neurological state assessed by the modified Rankin Scale (mRS) at discharge and during the 3-month follow-up period. The inclusion criteria for the study were age ≥18 years, clinical symptoms of stroke, absence of hemorrhage on initial cerebral imaging, and written consent to participate in the research. The study was conducted between March 2018 and July 2022 in 2 phases: 2018-2019 and 2021-2022, following ethical approval.

The study design aimed to include a minimum of 55 patients per group, resulting in a final population of 150 consecutive ischemic stroke patients admitted to a tertiary neurological hospital within 24 hours of symptom onset. All the patients underwent clinical assessments for stroke severity using the National Institutes of Health Stroke Scale (NIHSS) and radiological investigations using native cerebral CT or MRI. Standard laboratory parameters were recorded, and additional blood samples were collected to assess the baseline plasma levels of MMP-2 and MMP-9. A second head CT/MRI was performed during hospitalization for early detection of hemorrhagic transformation. Data on mortality rate and neurological functional state (evaluated by the mRS scale) at discharge and during the 3-month follow-up visit were comparatively analyzed. The collected data were analyzed using the R programming language, version 4.1.1. Group comparisons between the active and control groups were performed using Pearson's chi-squared test, Fisher's exact test, and a two-sample t-test. Correlation analysis was done using Pearson’s correlation coefficient analysis. The study variables were also explored through a univariate and multivariable regression model to find significant effects on patients’ outcomes. A p-value of <0.05 was considered statistically significant.

## RESULTS

### Demographic characteristics

The study cohort consisted of 150 acute ischemic stroke (IS) patients, with 55 patients developing hemorrhagic transformation (HT) during hospitalization, forming the active group. There were no significant differences in age, gender distribution, time to admission, or time to blood sample collection for MMPs measurement between patients in the active and control groups, as shown in Table 1. However, the stroke subtype, according to the TOAST criteria (Trial of ORG 10172 in Acute Stroke Treatment), showed significant differences between the groups (Table 1).

**Table 1:** Column I. Comparative general demographic data of the analyzed patients

Characteristic	Overall, N=150^1^	Control group, N=95	Active group, N=55	p-value^2^
**Age (years)**				0.51
Mean (SD)	71 (10)	71 (10)	70 (10)	
Median (IQR)	70 (12)	70 (14)	69 (10)	
Range	41 - 96	41 - 94	43 - 96	
**Gender**				0.14
Female	81 (54%)	47 (49%)	34 (62%)	
Male	69 (46%)	48 (51%)	21 (38%)	
**Time to admission (minutes)**				0.37
Mean (SD)	375 (318)	394 (340)	344 (278)	
Median (IQR)	240 (360)	240 (360)	240 (360)	
Range	60 - 1,440	60 - 1,440	60 - 1,200	
**Time to blood sample for MMPs (minutes)**				0.81
Mean (SD)	1,314 (632)	1,325 (496)	1,294 (817)	
Median (IQR)	1,320 (390)	1,350 (360)	1,320 (540)	
Range	120 - 5,760	120 - 2,880	180 - 5,760	
**Stroke subtype (TOAST)**				0.017
1 - large-artery atherosclerosis	54 (36%)	40 (42%)	14 (25%)	
2 - cardioembolism	63 (42%)	34 (36%)	29 (53%)	
3 - small vessel occlusion	7 (4.7%)	7 (7.4%)	0 (0%)	
4 - stroke of other determined etiology	18 (12%)	11 (12%)	7 (13%)	
5 - stroke of undetermined etiology	8 (5.3%)	3 (3.2%)	5 (9.1%)	
**Admission NIHSS score**				<0.001
Mean (SD)	13 (6)	11 (6)	15 (5)	
Median (IQR)	12 (9)	11 (8)	15 (6)	
Range	2 - 30	2 - 30	4 - 22	

**Table 1:** Column II. Comparative general demographic data of the analyzed patients

Characteristic	Overall, N=150^1^	Control group, N=95	Active group, N=55	p-value^2^
**Discharge NIHSS score**				<0.001
Mean (SD)	9 (6)	8 (5)	12 (5)	
Median (IQR)	8 (10)	7 (7)	12 (6)	
Range	0 - 24	0 - 24	2 - 21	
**Discharge mRS score**				<0.001
Mean (SD)	4 (1)	4 (1)	5 (1)	
Median (IQR)	4 (2)	4 (1)	4 (2)	
Range	1 - 6	1 - 6	2 - 6	
**3 months-follow-up mRS score**				<0.001
Mean (SD)	4 (2)	3 (2)	4 (2)	
Median (IQR)	3 (2)	3 (2)	4 (3)	
Range	0 - 6	0 - 6	2 - 6	

1. n (%); c("Mean (SD)", "Median (IQR)", "Range")

2. Pearson's Chi-squared test, Fisher's exact test, Two Sample t-test MMPs – matrix metalloproteinases

The simple logistic regression analysis revealed that HT was significantly higher in stroke type 2 (cardioembolic stroke) compared to type 1 (large-artery atherosclerosis stroke) (p=0.026) and in a stroke of undetermined origin (type 5) compared to type 1 (p=0.049).

Atrial fibrillation was significantly more common in the active group compared to the control group.

Atrial fibrillation was significantly more common in the active group compared to the control group (73% *versus* 47%, p=0.003). However, there were no significant differences between the two groups in terms of rates of other comorbidities such as diabetes, hypertension, myocardial infarction, previous stroke, obesity, smoking, and alcohol consumption.

The compared groups presented similar rates for the previous-to-stroke medication, including antiplatelets (active group 42%, control group 39%, p=0.73), anticoagulants (18% *versus* 11%, p=0.18), antihypertensives (82% *versus* 72%, p=0.39), oral antidiabetics (16% *versus* 18%, p=0.81), insulin (1.8% *versus* 7.4%, p=0.26), and statins (11% *versus* 7.4%, p=0.55).

The patients from the active cohort received either alteplase, either thrombectomy, or both (as bridging therapy) more frequently than the controls (24% *versus* 8.4%, p=0.01) and were admitted to the ICU unit more often (47% *versus* 18%, p<0.001), but with comparable ICU duration stay between groups (p=0.08).

Both admission and discharge NIHSS scores were significantly higher in the active group of IS patients compared to the control group (p<0.001), a situation reflected therefore in the discharge and 3 months follow-up outcomes, with significantly worse outcomes in the HT-group (p<0.001) (Table 1). Both the in-hospital mortality rate (33% *vs*. 18%, p=0.04) and the 3 months follow-up data were significantly higher in the active group (43% *vs*. 12%, p<0.001).

### Non-specific laboratory parameters profile and related data

In our research, there were no statistically significant differences in admission glycemia between the active and control groups. The mean admission glycemia was slightly elevated in the active group (8.0±0.41 mmol/l) compared to the control group (7.3±0.37 mmol/l), but the difference was not significant (p=0.26). Similarly, no significant differences were found in the coagulation panel results, including fibrinogen (p=0.21) and platelet count (p=0.45). However, the International Normalized Ratio (INR) values were significantly lower in the active group (1.27, range 0.89-2.00) than in the controls (1.38, range 0.94-2.28) (p=0.003).

The admission lipid profile analysis did not reveal any significant differences between the active and control groups (Table 2).

**Table 2:** Admission lipid profile of the studied patients

Characteristic	Overall, N=150^1^	Control group, N=95	Active group, N=55	p-value^2^
**Total cholesterol (mmol/l)**				0.94
Mean (SD)	5.42 (1.20)	5.42 (1.15)	5.40 (1.29)	
Median (IQR)	5.31 (1.60)	5.19 (1.43)	5.37 (1.90)	
Range	2.65 - 9.30	3.09 - 9.30	2.65 - 8.80	
**HDL-cholesterol (mmol/l)**				0.76
Mean (SD)	1.33 (0.34)	1.32 (0.30)	1.34 (0.40)	
Median (IQR)	1.27 (0.28)	1.26 (0.29)	1.30 (0.28)	
Range	0.96 - 3.76	0.96 - 2.70	1.00 - 3.76	
**LDL-cholesterol (mmol/l)**				0.99
Mean (SD)	3.30 (0.84)	3.30 (0.79)	3.30 (0.93)	
Median (IQR)	3.30 (1.10)	3.30 (1.00)	3.25 (1.50)	
Range	1.20 - 5.60	1.40 - 5.60	1.20 - 5.60	
**Triglycerides (mmol/l)**				0.78
Mean (SD)	1.76 (0.72)	1.77 (0.68)	1.74 (0.79)	
Median (IQR)	1.65 (0.71)	1.67 (0.70)	1.56 (0.74)	
Range	0.64 - 4.77	0.64 - 4.30	0.78 - 4.77	

1. n (%); c("Mean (SD)", "Median (IQR)", "Range")

2. Pearson's Chi-squared test, Fisher's exact test, Two Sample t-test INR – International Normalised Ratio, HDL – High-Density Lipoprotein, LDL – Low-Density Lipoprotein

**Figure 1:**
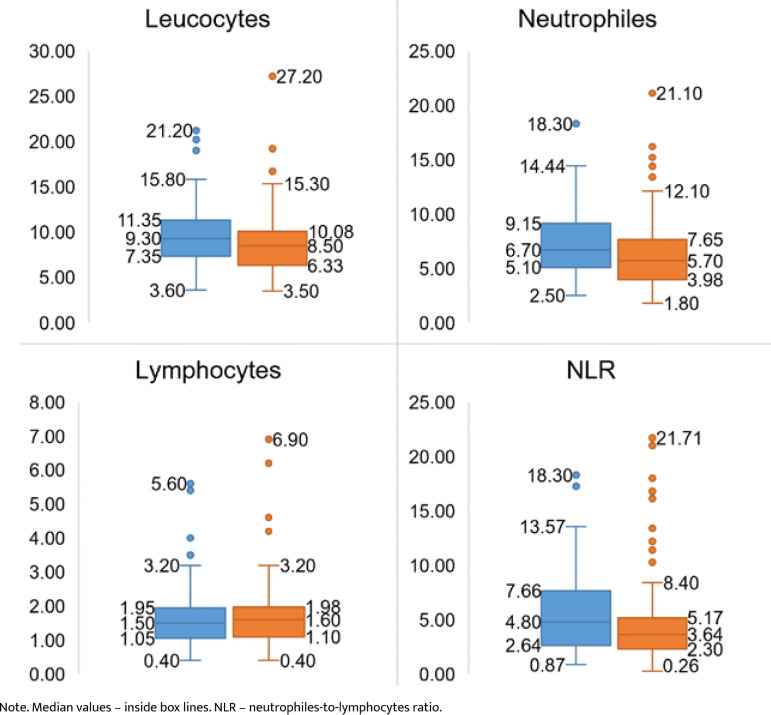
Comparison of admission inflammatory reaction parameters in the active (blue boxes) and control groups (orange boxes) of IS patients, 10^9^/l

For the inflammatory reaction study, we measured the total leukocyte count and the levels of neutrophils and lymphocytes upon admission. Additionally, we calculated a supplementary indicator called the neutrophil-to-lymphocyte ratio (NLR) by dividing the absolute values of neutrophils by lymphocytes (Figure 1).

For the absolute values, no significant differences were observed in the compared groups, with constant slightly higher numbers in the active group: total leucocytes (9.3*10^9^/l *vs*. 8.5*10^9^/l, p=0.082), neutrophils (6.7*10^9^/l *vs*. 5.7*10^9^/l, p=0.068), lymphocytes (1.7610^9^/l *vs*. 1.7*10^9^/l, p=0.75), and NLR (5.7 *vs*. 5.2, p=0.47).

The Pearson correlation analysis failed to prove a significant negative relation between NLR and HT risk (r(148)=0.05, p=0.46), mRS discharge score (r(148)=0.13, p=0.09), or the neurological status at the 3-month follow-up (by mRS score) (r(109)=0.09, p=0.34).

### Baseline MMP-2 and MMP-9 plasma level and clinical correlations

We initially performed a Pearson correlation analysis using non-standardized values of MMPs, for separated tranches, with the results indicating a negative, but statistically not significant, correlation between MMP-2, MMP-9 at admission, and the risk for HT (first tranche - MMP-2, r(78)=0.02, p=0.08, MMP-9, r(78)=0.1, p=0.4; second tranche – MMP-2, r(68)=0.21, p=0.08, MMP-9, r(68)=0.09, p=0.46).

**Figure 2:**
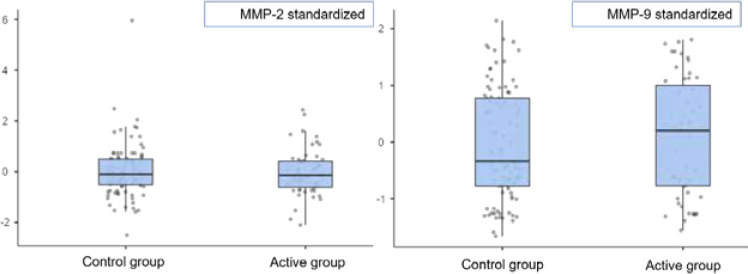
Comparison of standardized MMP-2 and MMP-9 levels in active and control groups (two samples t-test), ng/ml

Next, we converted the MMPs values from the two tranches into a new scale by standardizing the results and made a comparative analysis between the active and control group patients. The two-sample t-test for standardized values did not reveal any significant differences between the active and control groups for MMP-2 (p=0.72) and MMP-9 (p=0.37), as shown in Figure 2.

To study the correlation between the laboratory parameters and the risk for hemorrhagic transformation, we performed univariate and multivariate regression analyses (Table 3). To note, baseline INR was associated with a significantly lower risk for HT, both in the univariate (p=0.01) and multivariate analysis (p=0.03).

**Table 3:** Correlation between the laboratory parameters and the risk for haemorrhagic transformation among patients

Characteristic / type of regression analysis	Univariate analysis			Multivariate analysis		
	OR^1^	95% CI^1^	p-value	OR^1^	95% CI^1^	p-value
**Glycemia**	1.07	0.96, 1.19	0.23	1.14	0.96, 1.35	0.13
**Total cholesterol**	1.002	0.74, 1.35	0.99	1.05	0.37, 3.03	0.92
**LDL-cholesterol**	1.005	0.66, 1.53	0.98	1.07	0.27, 4.36	0.92
**Platelet count**	0.99	0.99, 1.003	0.51	0.99	0.99, 1.01	0.58
**INR**	0.11	0.02, 0.53	0.01	0.09	0.01, 0.75	0.03
**Leucocyte count**	1.07	0.98, 1.18	0.14	1.12	0.96, 1.3	0.15
**MMP-2**	0.99	0.69, 1.4	0.95	0.9	0.52, 1.47	0.68
**MMP-9**	1.14	0.8, 1.63	0.45	1.5	0.87, 2.62	0.15

1. OR = Odds Ratio, CI = Confidence Interval

## DISCUSSION

The aim of our research was to analyze non-specific laboratory data, including admission laboratory parameters, as well as baseline levels of MMP-2 and MMP-9 in the serum of patients with ischemic stroke. Our objective was to investigate the potential prognostic role of these parameters in relation to the risk of hemorrhagic transformation, discharge outcomes, and the 3-month follow-up status of the patients.

According to literature data, increased admission glycemia is associated with a higher HT rate and a worse neurological outcome in ischemic stroke patients [20]. Although the patients in the active group had slightly higher baseline glycemia, the difference did not reach the statistical threshold (p=0.26). Contrary to similar studies [21], the regression analysis results indicated that higher admission glycemia did not increase the risk for HT or poor neurologic outcomes at discharge and 3-month follow-up.

The hemostasis parameters play an important role in the evolution of IS patients, as numerous studies found that low platelet count and admission fibrinogen level correlate with the risk for HT [22]. Similarly, our analysis found slightly reduced plasma levels of both fibrinogen and platelets in the active group compared to the control group. In addition, the INR was also significantly reduced in the active group (p=0.003), contrary to literature data proving consistently that increased INR level is associated with a higher HT rate [23]. A possible explanation for this paradoxical result may be the association between a lower INR and the prothrombotic state with a subsequent bigger stroke in case of diminished INR baseline values, the stroke volume being an independent predictor of HT in ischemic stroke patients [24]. Studies on the correlation between admission INR and cerebral stroke volume found that lower INR was associated with larger strokes and worse outcomes [25, 26].

We also examined lipid profile parameters as non-specific factors in ischemic stroke patients. There is an ongoing debate in the literature, with studies indicating that lower total cholesterol levels and LDL-cholesterol are associated with a higher risk for HT [11, 27] and other studies proving the opposite [28]. Our research found that total cholesterol levels were higher in the active group, while LDL-cholesterol levels were similar in the compared levels. In our study, the lipidogram parameters did not increase the risk of HT or poor neurologic outcomes at discharge and the 3-month follow-up.

Among the non-specific laboratory factors, previous studies have suggested that inflammatory reaction parameters, such as increased baseline total leukocyte count and the neutrophils-to-lymphocytes ratio (NLR), hold promise for predicting adverse neurological outcomes, including symptomatic HT, in ischemic stroke patients [29–32]. However, our research did not find significant differences in the admission NLR values between the study groups (p=0.047). Furthermore, the Pearson analysis detected a negative correlation between NLR, HT, and stroke outcome, but these correlations did not reach statistical significance (p=0.46 for HT, p=0.09 for neurological discharge state, and p=0.34 for neurological recovery at 3 months follow-up). These findings differ from the results of similar studies [33], which may be attributed to the similar values of the studied parameters between the active and control groups (Figure 1).

Given the important role in the blood-brain barrier (BBB) integrity and function, matrix metalloproteinases represent attractive molecules for research in the context of ischemic stroke patients. Previous preclinical and clinical studies have shown promising results, suggesting that MMP-9 and MMP-2 may predict ischemic stroke outcomes, including hemorrhagic transformation (HT) and cerebral infarction volume [34, 35]. Consequently, we conducted a comparative analysis of plasma levels of MMP-2 and MMP-9 within 24 hours of admission in our patient cohort to highlight any potential correlations with the risk of HT and adverse neurological outcomes at discharge and during the 90-day follow-up.

While no significant differences were found for MMP-2 (p=0.72) and MMP-9 (p=0.37) between the studied groups, IS patients from the active cohort presented higher plasma levels of the analyzed MMPs. Moreover, the regression analysis found that increased admission levels of MMP-9 were associated with an elevated risk of hemorrhagic transformation (HT) and predicted worse neurological outcomes at discharge and during the 3-month follow-up period. However, the associations did not reach statistical significance. Increased MMP-2 levels were only correlated with worse discharge neurological states (Table 3).

Despite the abundance of research indicating a positive correlation between MMPs and HT, Cui *et al*. [36] also found lower MMP-2 levels in the HT group and no significant correlation between MMP-9 levels and the rate of HT, consistent with our study. Another possible explanation for the lack of significant correlations in our study could be attributed to the temporal expression patterns of MMPs [37] with early expression of MMP-2 (first hours) compared to MMP-9 (up to 48h). In our study, blood samples for MMP detection were collected at an average of 21.9 hours ± 51.6 minutes, with no significant differences between the groups (p=0.81). It is plausible that this collection time may have been too early to capture the maximum levels of MMP-9.

Our study has strengths, including a prospective design, a high number of variables analyzed, and multiple statistical methods applied to highlight the differences between the compared groups and underline possible correlations with outcomes in IS patients. Although corresponding to the study protocol, one important limitation of the actual research is the cohort size, as many positive/negative correlation results could not reach the significance threshold. Moreover, we could collect only one blood sample for MMPs analysis, and a dynamic analysis would be more informative in the settings of the variable temporal expression of MMP-2 and MMP-9.

## CONCLUSION

The hemorrhagic transformation had a significant negative impact on the outcome of ischemic stroke patients, both at discharge and during the 90-day follow-up. The analyzed laboratory data presented insignificant differences between the groups, except for the admission INR which had significantly lower values in the active cohort. However, further research with larger cohorts is necessary to identify specific laboratory biomarkers that can accurately predict hemorrhagic transformation and overall outcome in patients with ischemic stroke.
